# Exploring Sex Differences in the Relationship Between Emotion Regulation and Eating Disorders Symptoms During Early Adolescence

**DOI:** 10.3390/jcm15031237

**Published:** 2026-02-04

**Authors:** María Gámiz-Sanfeliu, Maria Fernández-Capo, Juliana Rojas-Rincón, Aikaterini Ampatzoglou, Cristina Fernández-Cardellach, Anna Garcia-Casanovas, Maite Garolera, Anna Carballo-Márquez, Bruno Porras-Garcia

**Affiliations:** 1 BrainXR Lab, Department of Psychology, Universitat Internacional de Catalunya, 08195 Sant Cugat del Vallès, Spainkaterinaampa@uic.es (A.A.); mgarolera@cst.cat (M.G.);; 2 Brain, Cognition, and Behavior Research Group, Consorci Sanitari de Terrassa-Hospital Universitari, 08227 Terrassa, Spain

**Keywords:** eating disorders, emotion regulation, rumination, early adolescence, sex differences

## Abstract

Difficulties in emotion regulation (ER) have been consistently associated with eating disorders (EDs). However, little is known about how this association operates during early adolescence, and the specific influence of sex. **Objectives:** This study aims to assess if maladaptive ER strategies predict greater ED symptomatology, while adaptive strategies predict lower levels of EDs symptoms among early adolescents. Additionally, the moderating effect of sex will also be assessed in these associations. **Method:** Ninety-eight Spanish-speaking adolescents aged 12–15 years (55 girls, 43 boys) participated in this study and completed a baseline assessment, including measures of EDs, adaptative (i.e., cognitive reappraisal) and maladaptive (i.e., expressive suppression and rumination) ER strategies. Independent sample *t*-tests were used to examine sex differences in age, ER, and ED symptoms. Hierarchical regression models assessed whether sex moderated the associations between ER strategies and ED symptoms. **Results:** Girls reported significantly higher levels of both brooding and reflective rumination compared to boys, but no sex differences were found in other measures. Regression analyses showed that expressive suppression and brooding rumination significantly predicted higher ED symptomatology, independent of sex. In contrast, cognitive reappraisal and reflective rumination were not associated with ED symptoms. No moderating effects of sex were observed in any model. **Conclusions:** Findings indicate that maladaptive ER strategies, particularly expressive suppression and ruminative brooding, predict greater ED symptom severity in early adolescence. However, sex did not moderate these relationships. These results underscore the importance of targeting maladaptive ER processes in adolescent prevention programs. Interventions focused on reducing maladaptive ER may be especially relevant at this developmental stage, when cognitive capacities for adaptive ER are still maturing.

## 1. Introduction

Eating disorders (EDs), characterized by dysfunctional eating behaviors and problematic weight control strategies, represent a significant global health concern, particularly among adolescents [1]. These disorders carry substantial medical, psychological, and social consequences, contributing to increased health care costs and placing considerable strain on families [2]. Over 80% of individuals affected by EDs do not receive treatment [3,4], underscoring the urgent need for early detection and preventive interventions. As such, the development and widespread implementation of effective prevention programs is a critical public health and research priority, especially during adolescence, a developmental period marked by heightened vulnerability to the onset of EDs [5].

Theoretical frameworks increasingly emphasize the central role of affective processes in the etiology and maintenance of EDs, suggesting that the interplay between cognitive and emotional mechanisms is key to understanding and addressing these disorders [6,7,8]. This perspective is reinforced by several studies showing that affective states, and particularly the emotion regulation (ER) cycle, are instrumental to ED onset and maintenance [7]. ER is a multifaceted construct that has received substantial attention in psychological research, particularly due to its critical role in psychological adjustment and its implications across a wide range of psychopathologies [9,10]. ER is generally defined as a set of processes, both automatic and deliberate, by which individuals influence the emotions they experience, when they occur, and how they are subjectively experienced and behaviorally expressed [11]. It is also a dynamic and flexible process that can occur at multiple stages of the emotional response, before, during, or after an emotion arises [12,13]. ER strategies include attentional control, emotional awareness, cognitive reappraisal, problem solving, and response modulation. When used adaptively, such strategies enhance coping, emotional resilience, and psychological well-being, aligning with broader models that emphasize the coordinated role of attention, cognition, and behavioral regulation in managing emotions [14]. In Gross’s process model, regulation can occur at multiple points in the unfolding emotion trajectory, spanning different strategies, such as cognitive change (e.g., cognitive reappraisal), and response modulation (e.g., expressive suppression) [8,11]. A key implication is that strategies differ not only in content but in timing: antecedent-focused strategies (e.g., reappraisal) act earlier and may alter the emotional response before it is fully generated, whereas response-focused strategies (e.g., suppression) act later and may reduce observable expression without necessarily reducing subjective experience and may even increase physiological responding [11]. In addition, the process model emphasizes that the adaptiveness of ER is context-dependent, such that the same strategy can be more or less effective depending on situational demands and regulatory goals [11,13].

Difficulties in ER, often referred to as emotion dysregulation, have been consistently associated with a wide range of psychopathological outcomes, including mood disorders, anxiety, borderline personality disorder, and EDs [9,15]. Emotion dysregulation refers to deficits in the capacity to monitor, evaluate, and modify emotional responses in a goal-directed and context-appropriate manner [16]. Individuals experiencing such difficulties may struggle to shift attention away from distressing stimuli, regulate behavioral impulses, or employ adaptive strategies to modulate negative affect [8,17]. Individuals with EDs often rely on maladaptive ER strategies, to cope with distressing affective states [6,7,18,19]. These strategies, rather than reducing distress, tend to reinforce and intensify disordered eating behaviors. For instance, ED symptoms can become negatively reinforced when they provide short-term relief from distress, increasing reliance on maladaptive regulation patterns over time [20]. One illustrative pathway consistent with the model is that suppression may leave distress insufficiently resolved (or amplify regulatory load), increasing reliance on secondary maladaptive processes (e.g., perseveration/rumination) and symptom-related coping, whereas reappraisal may buffer the impact of stressors on eating-related outcomes [21,22]. As such, previous studies found that individuals with EDs reported greater difficulties in core adaptative ER domains, including emotional awareness, clarity, acceptance, and emotional impulse control, as compared to healthy controls [23,24]. A network metanalysis found that rumination and expressive suppression were the ER strategies most strongly associated with ED symptoms, whereas deficits in cognitive reappraisal were less closely related [21]. These findings align with ecological momentary assessment studies showing that increases in repetitive negative thinking predict real-time engagement in behaviors such as binge eating and body checking [25].

Furthermore, adolescents may be particularly at risk, as their ER capacities are still developing, making them more susceptible to maladaptive coping mechanisms [26]. In fact, ER develops progressively from childhood into adolescence, closely tied to neurobiological maturation processes that influence physiological, cognitive, and behavioral systems [27]. From a developmental psychopathology perspective, early adolescence is a particularly sensitive period in which changing socio-emotional demands interact with still-maturing emotion-regulatory systems, shaping risk and resilience trajectories [28]. Compared with later adolescence and adulthood, early adolescence is characterized by heightened socio-emotional reactivity alongside ongoing maturation of cognitive control, which can make the deployment of effortful strategies more variable and less consistent [29]. At a neurodevelopmental level, brain systems implicated in affect generation and control, particularly limbic regions and the prefrontal cortex, undergo structural and functional changes across adolescence, supporting gradual improvements in the capacity to modulate emotional responses [25,30]. Accordingly, ER capacities tend to strengthen from early to later adolescence, including increases in the use and effectiveness of cognitively demanding strategies such as cognitive reappraisal as regulatory control consolidates [31,32]. Supporting this perspective, previous research indicates that younger adolescents (ages 9–12) scored lower in the use of ER strategies compared to older adolescents (ages 13–16), with girls generally reporting more frequent use of strategies, both adaptive (e.g., cognitive reappraisal) and maladaptive (e.g., rumination), in response to negative emotions such as sadness, anxiety, and anger [27]. Although girls tend to endorse a broader range of ER strategies, studies suggest that maladaptive strategies such as rumination and emotion suppression are more strongly associated with psychological distress and depressive symptoms than the protective effects offered by adaptive strategies [33]. These sex differences in ER may be particularly relevant in the context of EDs, which show a higher prevalence among females and typically emerge during adolescence. Girls’ increased use of maladaptive ER strategies, especially rumination, may increase vulnerability to ED symptomatology, contributing to the onset and persistence of behaviors such as restrictive eating, binging, and purging [6,7,26]. Notably, while girls may show greater ER engagement during early adolescence, some evidence suggests this advantage diminishes or reverses in later adolescence, indicating an interaction between age and sex in the development of ER abilities [27].

Taken together, existing findings highlight the developmental sensitivity and sex-specific patterns of ER, as well as their critical relevance to the etiology and maintenance of EDs. Although previous research has explored the role of ER in EDs during adolescence, it remains a pressing need for more exhaustive analyses that specifically address sex differences in these associations, an area that has received relatively limited attention to date [21]. Moreover, most studies have concentrated on adolescence and adulthood (e.g., [19,23]), leaving a significant gap in our understanding of how ER difficulties relate to ED symptoms during early adolescence. While a few studies have included younger participants (e.g., [27]), the specific interplay between ER and ED symptomatology in early adolescence remains relatively underexplored. Addressing this gap is essential for identifying early risk trajectories and optimizing the timing and content of preventive interventions in EDs, particularly for adolescents [34].

The present study will examine the relationship between ER strategies and ED symptoms in a community sample of healthy early adolescents. Specifically, we will assess whether adaptive ER strategy, such as cognitive reappraisal, and maladaptive strategies (i.e., expressive suppression and rumination) predict greater or lower family-reported ED symptomatology. In addition, we will explore whether these associations differ by sex, comparing patterns between young girls and boys. Based on prior research, we expect that greater use of maladaptive ER strategies will predict greater levels of ED symptoms [6,18,19,21]. Second, we hypothesize that greater use of cognitive reappraisal will predict lower ED symptoms [9,26]. Lastly, we expect these associations to be stronger among girls than boys, reflecting sex differences in both adaptative and maladaptive ER strategies [27].

## 2. Materials and Methods

### 2.1. Participants

Ninety-eight Spanish-speaking adolescents (55 girls and 43 boys) were recruited from four schools in the metropolitan area of Barcelona (Catalonia). All participants were 12–15 years old. Prior to enrollment, the adolescents and their legal guardians received information about the study and provided written informed consent. Eligibility was restricted to students who could read and understand Spanish, because all evaluations and questionnaires were administered in Spanish. Adolescents were excluded if they presented or their legal guardians reported severe psychiatric symptoms (e.g., manic episodes or psychosis), serious neurodevelopmental disorders (such as severe autism spectrum disorder or significant intellectual disability), or any major physical, motor or sensory impairments that would hinder completion of the study assessments. A current or past self-reported or family history of an ED was also considered for exclusion; nevertheless, none of the adolescents or their families reported such a diagnosis.

### 2.2. Measures

All assessments included Spanish-validated and translated questionnaires adapted to be used among adolescents from secondary school.

The SENA (Sistema de Evaluación de Niños y Adolescentes [35]) comprises nine questionnaires tailored to three age groups: Infant (3–6 years), Primary (6–12 years), and Secondary (12–18 years). These instruments were administered across key developmental contexts (e.g., family and school) and include three corresponding self-report models based on the child’s age. Each questionnaire employs a multidimensional framework, organizing content into three categories: internalizing problems, externalizing problems, and a set of specific issues, one of which is the Eating Behavior Problems scale. In this study, only data from the Eating Behavior Problems scale were used. This scale contains 10 items, each rated on a 5-point Likert scale ranging from 1 (“never or almost never”) to 5 (“always or almost always”) and focuses on assessing ED symptoms across a continuum of severity. The Eating Behavior Problems scale has demonstrated excellent psychometric properties, with Cronbach’s alpha values ranging from 0.81 to 0.84 in both clinical and nonclinical adolescent samples [35].

The Emotion Regulation Questionnaire for Children and Adolescents (ERQ-CA) assesses ER strategies. This 10-item, self-report instrument has a 5-point Likert scale, with a total score ranging from 10 to 50, with higher scores reflecting more frequent use of the targeted strategies. Two subscales comprise the ERQ-CA: Cognitive Reappraisal, characterized by reframing emotion-eliciting situations to alter their impact, and Expressive Suppression, characterized by inhibiting ongoing emotional expressions. The ERQ-CA has demonstrated sound psychometric properties, including high internal consistency (α = 0.79 for cognitive reappraisal; *α* = 0.73 for expressive suppression), adequate three-month test–retest reliability (*r* = 0.69 for both subscales), and strong convergent and discriminant validity in samples spanning multiple age groups [36,37]. The Spanish adaptation of this measure was used for this study, which has also been validated among early adolescents, showing adequate internal consistency and convergent validity [38].

The Rumination Response Scale—Short form (RRS-SF; [39]) is a self-report measure designed to assess ruminative thought processes, specifically brooding and reflection. In this study, the Spanish version of the scale was used [40]. The RRS consists of 10 items rated on a 4-point scale ranging from 1 (“almost never”) to 4 (“almost always”). The scale includes two subscales: Brooding (e.g., “How often do you think, ‘Why can’t I handle things better?’”) and Reflection (e.g., “How often do you write down what you are thinking and analyze it?”). Both subscales were used for this study. The Spanish version of the RRS has demonstrated excellent internal consistency and adequate temporal stability in Spanish-speaking samples. Specifically, the Spanish RRS–Short Form showed good reliability (*α* = 0.86) in Spanish high school and college students [41]. While the Spanish adaptation of the full RRS also showed very high internal consistency (*α* = 0.93) alongside adequate test–retest reliability (*r* = 0.56) [40].

### 2.3. Procedure

This was a cross-sectional study conducted between January 2024 and June 2025, which belonged to the baseline assessment of a greater preventive project focused on ER, general mental health and adolescence. This broader project has been registered in clinicaltrials.gov (NCT06236919). The research followed ethical guidelines and was approved by the corresponding author’s university institutional review board (Ref. PSI-2023-04). Before participation, potential participants and their legal guardians were fully informed about the project’s objectives, procedures, and confidentiality measures. Written informed consent was obtained from legal guardians, while students gave their oral assent.

Four secondary schools from the metropolitan area of Barcelona participated in the study. The initial contact with families of potential participants was established through informational online presentations conducted at the participating schools. These sessions provided a comprehensive overview of the study, including its objectives, procedures, and the importance of participation. Families had the opportunity to ask questions, and printed informational materials were distributed to ensure that they had sufficient details before deciding on participation. In contrast, face-to-face meetings were held with school staff and students during school hours to further explain the study and answer any questions. The school counseling staff collaborated closely with the research team to maintain communication with families, address concerns, and support the recruitment process.

The recruitment process consisted of a two-step screening procedure conducted by a member of the research team and school counselors. In the first step, potential participants were identified based on their school enrollment and age group. Only students enrolled in first and second years of secondary mandatory education according to the Spanish education system (equivalent to 12–15 years old) were considered for participation, with a pool of 519 students in the four schools. In the second step, those who met the inclusion criteria and expressed interest in participating, 98 students, were encouraged to discuss the study with their parents or guardians. Once all parental consent and student assent were obtained, participants were officially enrolled. Then, students attended the baseline assessment session during regular school hours, which was conducted in a classroom or a designated quiet area within their schools. A member of the research team supervised the session, provided standardized instructions, and ensured that students completed the assessments under the same conditions. The assessment battery included all measures described in Section 2.2, along with other mental health and cognitive functioning measures. Regarding the SENA, it was distributed in a paper-based format to families and tutors to gather additional perspectives on overall externalizing/internalizing symptoms, including the ED subscale used in this study.

### 2.4. Data Analysis

SPSS (v.24) was used for all statistical analyses and graphs. Independent sample *t*-test analyses were run to assess sex differences in age, ER strategies and ED symptoms. In addition, moderated simple regression analyses were conducted to examine the moderating effect of sex (girls/boys) on the relationship between ER strategies and ED symptoms. Two hierarchical models were tested for each predictor (i.e., Expressive suppression, cognitive reappraisal, and brooding and reflective subtypes of rumination): Model 1 included the main effects of each predictor and moderator (sex); Model 2 added the interaction term (predictor * sex) to examine moderation. Effect sizes were examined by assessing *R*^2^ statistic to determine the additional explanatory power of the interaction term. All statistical analyses were conducted at a two-tailed significance level of *p* < 0.05. Before running the regression analyses, key statistical assumptions were examined to ensure the validity of the models. Linearity was established by visual inspection of scatterplots, and there was no evidence of multicollinearity. Although three outliers were identified, none were deemed to need removal since results were not significantly modified by including them. Lastly, most studentized residuals were normally distributed, as assessed by Shapiro–Wilk’s test (*p* > 0.05).

## 3. Results

Independent sample *t*-tests showed no significant sex differences in age, ED symptomatology, or the use of expressive suppression and cognitive reappraisal strategies. However, significant differences emerged in rumination tendencies. Adolescent girls reported higher levels of brooding compared to boys, and also higher levels of reflection than boys (see Table 1).

### 3.1. Relationship Between ER (Expressive Suppression and Cognitive Reappraisal) and EDs as Moderated by Sex

**Figure 1:**
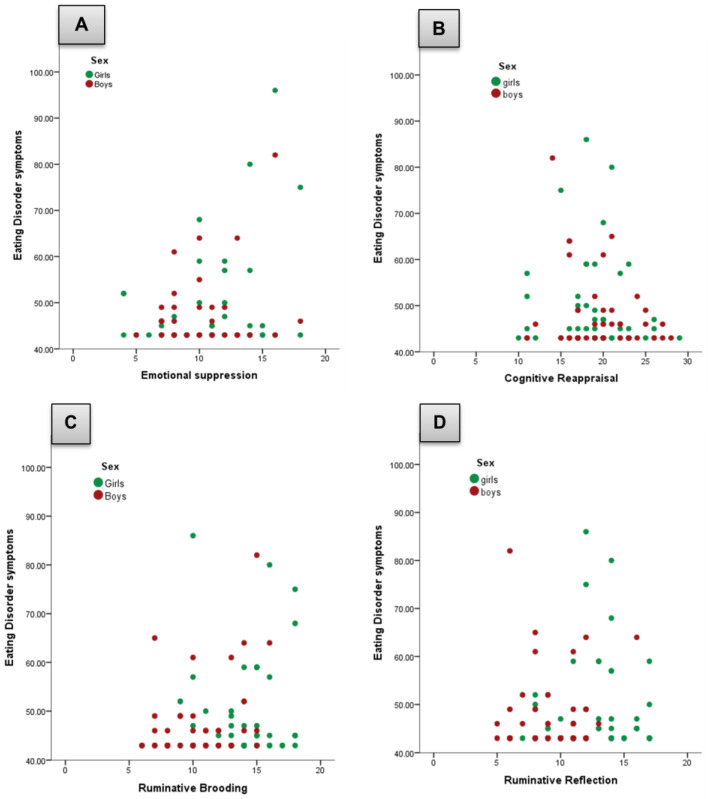
(**A**–**D**) Scatter plots displaying the relationship between emotion regulation strategies and eating disorder symptoms, moderated by sex.

Results from hierarchical regression models showed that the interaction between emotion suppression and sex did not significantly predict ED symptoms, with the inclusion of the interaction term explaining only an additional 2% of the variance, *F*(1, 94) = 0.157, *p* = 0.691. Consequently, the interaction term sex was removed from the model. The final model revealed that expressive suppression significantly predicted ED symptoms, *F*(1, 94) = 3.947, *p* = 0.023, explaining 5.7% of the variance. A statistically significant positive association between emotion suppression and ED symptoms, *b* = 0.768, *SE* = 0.274, showed that higher emotion suppression scores were associated with more severe ED symptoms, irrespective of sex (see Figure 1A).

Regarding cognitive reappraisal, there was not a significant moderation effect of sex and the addition of the interaction term accounted for only 3% of the variance, *F*(1, 94) = 0.297, *p* = 0.587. Therefore, the interaction term was also removed from the final model. In this revised model, cognitive reappraisal did not also predict ED symptoms, *F*(1, 94) = 1.382, *p* = 0.256, across sexes (see Figure 1B).

### 3.2. Relationship Between ER (Brooding and Reflection Rumination Subtypes) and EDs as Moderated by Sex

For brooding rumination, results from the hierarchical regression models did not show a significant moderation effect of sex (*p* = 0.693), so the interaction term was removed. However, with the revised model, brooding marginally predicted ED symptoms, *F*(1, 94) = 3.300, *p* = 0.053, explaining 4% of the variance. A positive relationship emerged between brooding and ED symptoms, *b* = 0.734, *SE* = 0.300, indicating that higher levels of brooding predicted greater ED symptomatology regardless of sex (see Figure 1C). In contrast, the reflective subscale did not show neither a significant moderation effect of sex (*F*(1, 94) = 0.358, *p* = 0.700, *R*^2^ = 0.007) nor a main effect of reflective rumination on ED symptoms (*F*(1, 94) = 0.331, *p* = 0.566, *R*^2^ = 0.011) among girls and boys (see Figure 1D).

## 4. Discussion

The present study examined the associations between several ER strategies, including cognitive reappraisal, expressive suppression, and general rumination, with ED symptomatology in a community sample of early adolescents, while also exploring the moderating role of sex. Several notable findings emerged. Expressive suppression significantly predicted ED symptom severity across sexes, while cognitive reappraisal did not predict ED symptoms. General brooding rumination also significantly predicted ED symptoms, whereas reflective rumination was not, in both sexes again. These results partially supported our hypotheses, as maladaptive ER strategies, particularly suppression and brooding significantly predicted greater ED symptomatology, while cognitive reappraisal did not exhibit the expected protective association.

Expressive suppression predicted greater risk of ED symptom development among both girls and boys. These findings are consistent with studies in adults with EDs showing that frequent use of expressive suppression is associated with increased binge eating episodes, heightened body dissatisfaction, and other ED symptoms [26,30]. Similar patterns have been observed in adolescent populations, where expressive suppression predicts the exacerbation of restrictive eating patterns and compensatory behaviors, often serving as a maladaptive mechanism for regulating negative affect [42]. However, relatively few studies, such as the present one, have focused specifically on this relationship during early adolescence, a developmental stage characterized by heightened emotional reactivity and ongoing maturation of neural systems involved in cognitive control. Neurodevelopmental evidence shows that during this period, subcortical limbic structures (e.g., amygdala, striatum) are highly reactive to emotionally salient stimuli, while prefrontal cortical regions that support executive functioning and flexible ER are still undergoing structural and functional refinement [43]. This developmental imbalance makes it more difficult for early adolescents to modulate emotions adaptively, leading to a greater reliance on automatic or less cognitively demanding strategies such as expressive suppression [44]. Furthermore, adolescents who rely more often on expressive suppression exhibit greater increases in internalizing symptoms over time [45].

Expressive suppression is considered a maladaptive ER strategy because it targets only the outward expression of emotions rather than addressing or modifying the underlying emotional experience [11]. This mismatch between inner experience and outward expression can lead to several processes that directly increase vulnerability to ED symptoms. First, the rebound effect of expressive suppression often intensifies and prolongs negative emotions such as shame, anxiety, and sadness, which are known triggers for disordered eating behaviors [9,30,46]. For example, adolescents who suppress distress may experience heightened emotional discomfort and use behaviors such as binge eating or dietary restriction to temporarily relieve or control these intensified feelings [26]. Expressive suppression also increases physiological arousal (e.g., elevated heart rate, muscle tension), which can be particularly aversive for adolescents who may have difficulty identifying or tolerating these internal states [44]. These difficulties are especially pronounced among adolescents at risk for EDs, who consistently demonstrate lower levels of emotional awareness and interoceptive accuracy compared to their peers [47,48]. This reduced emotional awareness may amplify the discomfort associated with heightened physiological arousal, further driving reliance on maladaptive eating behaviors. Furthermore, because suppression conceals outward emotional expressions, it makes it less likely that others will recognize when support is needed [48]. This reduced access to social support can lead to increased feelings of isolation and loneliness, which has been strongly associated with the development of EDs [49,50].

Contrary to our expectations, cognitive reappraisal did not show a significant association with ED symptomatology in our early adolescent sample. While some studies in adolescent and adult populations have found that higher use of cognitive reappraisal is associated with lower levels of psychopathology (e.g., [9]), other research aligns with our findings. A network meta-analysis highlighted that maladaptive ER strategies, particularly rumination and emotional non-acceptance, are more strongly associated with ED symptoms than deficits in cognitive reappraisal, suggesting that the latter may play a less central role in ED vulnerability [21]. These findings may be explained by the fact that cognitive reappraisal requires relatively sophisticated executive functioning and cognitive flexibility, capacities still under development in early adolescence [27,51]. In emotional situations related to EDs, cognitive reappraisal may be too complex, effortful or under-used [30]. Adolescents may therefore default to more automatic or less cognitively demanding strategies such as expressive suppression or rumination.

Our findings also underscore the importance of distinguishing between subtypes of rumination as predictors of ED symptomatology. Specifically, general brooding rumination did significantly predict greater ED symptom severity, whereas reflective rumination did not. This pattern is consistent with previous research demonstrating that brooding, a passive and judgmental focus on the causes and consequences of negative affect, is more strongly linked to psychopathology than reflection [42]. Brooding has been shown to amplify negative affect and foster maladaptive coping responses, thereby perpetuating emotional dysregulation [52]. In the context of EDs, brooding may involve perseverative self-critical thoughts about body image, eating behaviors, or perceived failures in self-control, which may reinforce cycles of dietary restriction, binge eating, or compensatory behaviors [53]. In contrast, reflective rumination in early adolescence does not yet function as a protective factor because metacognitive and executive control capacities are still developing during this period [27]. As discussed before, these neurodevelopmental constraints can limit the effectiveness of more cognitively demanding strategies, particularly when adolescents are confronted with emotionally charged ED-related situations. Reflective rumination during this developmental window may therefore fail to provide sufficient regulation and could even perpetuate ED-related preoccupation rather than facilitating adaptive problem-solving [21]. The divergence between these two forms of rumination may also reflect their different behavioral correlations, in which brooding is more strongly associated with avoidance and emotional inertia, whereas reflection has been associated with more active coping strategies in older adolescent populations [52]. Future research should examine whether the functional distinction between brooding and reflection becomes more pronounced later in adolescence, as ER capacities mature, and whether interventions can be designed to reduce brooding while simultaneously promoting adaptive reflective processing.

Even though girls reported significantly higher levels of brooding rumination and marginally greater expressive suppression than boys, sex did not moderate the associations between these ER strategies and ED symptoms. Consistent with our findings, recent evidence regarding sex/gender differences in ER–ED associations still remains mixed [54,55]. For instance, a recent meta-analysis also found that brooding predicted disordered eating in adolescents and young adults regardless of gender, although the strength of the association was somewhat greater in females [55]. Similarly, brooding and emotional non-acceptance were identified as central nodes in ED psychopathology networks, surpassing cognitive reappraisal or reflective rumination in predictive weight, and noted that these associations remain significant across sexes [21].

A plausible interpretation concerns developmental timing. Early adolescence may be a period in which affective reactivity and ER constraints are elevated across sexes, whereas sex-differentiated trajectories on the relationship between ER and ED symptoms may become more pronounced later in adolescence as pubertal and social transitions unfold [28,29]. In parallel, gendered sociocultural pressures may shape the prevalence of certain strategies, particularly rumination among girls [27,56], without necessarily altering their functional relation to symptom expression in early adolescence, especially in community samples with relatively low symptom severity. Consistent with this interpretation, other reviews have suggested that rumination may operate as a mediating mechanism in the relationship between sex/gender and disordered eating, which would yield sex differences in mean brooding levels without requiring a sex-by-rumination interaction [54]. Moreover, moderation may be contingent on the specific symptom domain (e.g., binge-eating frequency versus global ED symptoms) and may therefore be obscured when broad ED symptoms are assessed, particularly given concerns that commonly used ED measures may be less sensitive to male-typical symptom presentations [54]. Finally, expressive suppression, as a response-focused strategy closely aligned with experiential avoidance, is associated with increased sympathetic activation and regulatory costs that do not appear to be sex-specific [11]. For instance, suppression induces similar sympathetic arousal (e.g., elevated heart rate, muscle tension) regardless of sex [11].

The present findings have several implications for the prevention and early identification of ED symptomatology. By demonstrating that expressive suppression and the brooding subtype of rumination predict ED symptoms in early adolescence, this study underscores the clinical relevance of targeting maladaptive ER processes within universal prevention initiatives. Given that these strategies are modifiable and amenable to intervention [57], prevention efforts should incorporate ER skill-building components aimed at reducing reliance on suppression and maladaptive rumination, particularly in younger adolescents at risk [58]. Such content could be embedded within the school curriculum through structured socio-emotional learning modules that strengthen emotional awareness and labeling, promote acceptance-based responses to aversive affect, enhance distress-tolerance skills, and foster adaptive help-seeking and emotion communication with peers and caregivers. For adolescents presenting elevated brooding or early ED concerns, a stepped-care framework within schools (e.g., targeted small-group sessions and referral pathways) could incorporate selective/indicated modules addressing perseverative negative thinking (e.g., rumination-focused cognitive-behavioral techniques), consistent with evidence that rumination is modifiable in youth [59]. In addition, integrating Dialectical Behavior Therapy (DBT)-informed skills training in school-based prevention (e.g., mindfulness, distress tolerance, and emotion regulation modules) may be particularly well aligned with our findings, as these components directly target maladaptive ER processes implicated in ED-related distress and have been increasingly applied to ED prevention and early intervention [60]. More broadly, these results are informing ongoing efforts from our research group, to develop and evaluate new school-based prevention approaches that pair ER skills training with engaging delivery formats, such as extended reality (XR) technology, as a potential avenue to enhance engagement and adherence in preventive school-based interventions [61,62].

These implications also highlight the importance of developmental timing: early adolescence constitutes a sensitive window for both the consolidation of ER capacities and the emergence of ED risk factors, such that intervening during this period may help prevent maladaptive emotional processes from becoming entrenched. Finally, the absence of sex moderation in the ER–ED associations suggests that these ER-focused approaches are likely to be beneficial across sexes; nevertheless, prevention programs may consider gender-responsive emphasis on brooding reduction, given its higher endorsement among girls [27,55] and in the present sample.

Despite its contributions, this study has several limitations that should be considered. First, the cross-sectional design prevents the determination of stronger causal relationships between ER strategies and ED symptomatology. While we found significant associations, it is unclear whether maladaptive ER strategies precede and contribute to ED symptoms or whether existing ED symptoms exacerbate reliance on these strategies. Future research should therefore employ multi-wave longitudinal designs (e.g., prospective cohorts with cross-lagged or transactional models) to test whether ER predicts subsequent changes in ED symptoms, whether ED symptoms predict changes in ER, or whether bidirectional effects are present. Second, although we examined several core ER strategies, the study relied exclusively on self-report questionnaires administered in a single assessment session. This may limit the precision with which dynamic ER processes were captured, and responses may be influenced by recall biases. Furthermore, the measures used do not capture the situational flexibility with which ER strategies are deployed, a factor increasingly recognized as central to adaptive emotional functioning [63]. Incorporating real-time assessment methods such as ecological momentary assessment could provide a more nuanced understanding of how the relationship between ER strategies and ED symptoms operates in daily life. Third, ED symptoms were assessed using the SENA Eating Behavior Problems scale, which is based on parent-report rather than adolescent self-report. Although this decision was appropriate given participants’ age, parent informants may have limited access to more internal ED-related experiences (e.g., weight/shape concerns, guilt, preoccupation), potentially leading to under-detection of subjective symptomatology and attenuated associations. Future studies should therefore replicate these findings using validated adolescent self-report instruments alongside parent-report to enhance comparability across studies and improve ecological validity. In addition, incorporating multi-informant assessments (e.g., school tutors and, where appropriate, peers) and context-sensitive methods could better capture ER and eating-related difficulties as they manifest in school and other everyday settings. Fourth, the sample was relatively small and drawn from four private or concertate schools in a single metropolitan area, which may limit generalizability. Because participants were recruited from a single metropolitan area in Spain, generalizability may be limited, as cultural norms can shape the use and social consequences of ER strategies (e.g., suppression) [64] and socioeconomic risk is linked to variability in adolescents’ ER development [65,66]. Future studies should replicate these findings in larger and more diverse samples, including participants from different socioeconomic strata, educational settings, and cultural contexts, will be important to establish the broader applicability of the results. Fifth, while the moderating role of sex was examined, we did not explore gender identity or consider sociocultural factors (e.g., exposure to appearance ideals, peer and family influences) that could interact with ER processes in shaping ED risk. Finally, other potentially relevant ER strategies such as acceptance, problem-solving, and avoidance were not assessed. Expanding the range of ER constructs examined and using methods that can capture contextual variability will be essential for refining theoretical models of ED risk and informing the design of universal or selective prevention programs in this area.

## 5. Conclusions

In conclusion, our findings highlight a consistent pattern across sexes. Expressive suppression emerged as a significant predictor of greater ED symptom severity, whereas cognitive reappraisal was not associated with ED symptoms. Similarly, brooding rumination, but not reflective rumination, was robustly associated with higher ED symptomatology for both girls and boys. Taken together, these results provide partial support for our hypotheses and underscore the relevance of maladaptive ER strategies, particularly suppression and brooding, high risk factors of developing elevated ED symptoms in early adolescence.

## Figures and Tables

**Table 1:** Sex differences in age, ER strategies and ED symptoms.

	Adolescent Girls (*n* = 55)	Adolescent Boys (*n* = 43)		
	Mean (*SD*)	Mean (*SD*)	*t*	*p*
Age (years)	12.84 (0.63)	12.76 (0.88)	0.469	0.640
SENA ED	48.43 (9.67)	47.97 (8.29)	0.248	0.804
ERQ-CA Expressive suppression	11.36 (3.38)	10.30 (3.11)	1.595	0.114
ERQ-CA Cognitive reappraisal	19.11 (3.96)	19.86 (4.18)	−0.951	0.365
RRS-SF Brooding	13.05 (3.26)	10.60 (2.67)	3.986	<0.001
RRS-SF Reflection	12.90 (3.47)	9.40 (2.78)	4.861	<0.001

SENA ED = Eating Disorder Problems scale from the Sistema de Evaluación de Niños y Adolescentes (SENA); ERQ-CA = Emotion Regulation Questionnaire for Children and Adolescents; RRS-SF = Rumination Response Scale—Short Form.

## Data Availability

The datasets generated and/or analysed during the current study are available from the corresponding author upon reasonable request.
